# Integrins and ERp57 Coordinate to Regulate Cell Surface Calreticulin in Immunogenic Cell Death

**DOI:** 10.3389/fonc.2019.00411

**Published:** 2019-05-28

**Authors:** Chi-Chao Liu, Pascal Leclair, Foujan Pedari, Heidi Vieira, Mahdis Monajemi, Laura M. Sly, Gregor S. Reid, Chinten James Lim

**Affiliations:** ^1^Department of Pediatrics, University of British Columbia, Vancouver, BC, Canada; ^2^Michael Cuccione Childhood Cancer Research Program, BC Children's Hospital Research Institute, Vancouver, BC, Canada

**Keywords:** integrins, calreticulin, ERp57, immunogenic cell death, leukemia, cell adhesion

## Abstract

Therapy-induced presentation of cell surface calreticulin (CRT) is a pro-phagocytic immunogen beneficial for invoking anti-tumor immunity. Here, we characterized the roles of ERp57 and α-integrins as CRT-interacting proteins that coordinately regulate CRT translocation from the ER to the surface during immunogenic cell death. Using T-lymphoblasts as a genetic cell model, we found that drug-induced surface CRT is dependent on ERp57, while drug-induced surface ERp57 is independent of CRT. Differential subcellular immunostaining assays revealed that ERp57^−/−^ cells have minimal cytosolic CRT, indicating that ERp57 is indispensable for extra-ER accumulation of CRT. Stimulation of integrin activity, with either cell adhesion or molecular agonists, resulted in decreased drug-induced surface CRT and ERp57 levels. Similarly, surface CRT and ERp57 was reduced in cells expressing GFFKR, a conserved α-integrin cytosolic motif that binds CRT. Drug-induced surface ERp57 levels were consistently higher in CRT^−/−^ cells, suggesting integrin inhibition of surface ERp57 is an indirect consequence of α-integrin binding to CRT within the CRT-ERp57 complex. Furthermore, β1^−/−^ cells with reduced expression of multiple α-integrins, exhibit enhanced levels of drug-induced surface CRT and ERp57. Our findings highlight the coordinate involvement of plasma membrane integrins as inhibitors, and ERp57 originating from the ER as promoters, of CRT translocation from the ER to the cell surface.

## Introduction

Immunogenic cell death (ICD) is a form of therapy-induced tumor cell death which culminates in the release of damage associated molecular patterns (DAMPs) able to invoke an effective antitumor immune response (1, 2). Certain ICD-inducers able to provoke endoplasmic reticulum (ER) stress result in cell surface presentation of several ER-resident chaperone proteins as DAMPs, including calreticulin (CRT, encoded by *CALR*) and ERp57 (encoded by *PDIA3*) (3, 4). Even though surface ERp57 is non-immunogenic, it is non-dispensable for presentation of surface CRT as the immunogenic counterpart (4, 5). Thus, a mechanistic understanding of the regulators of surface CRT can improve the efficacy of therapies that optimize the ICD response.

CRT is a Ca^2+^ binding and buffering lectin that is highly abundant in the ER-lumen (6, 7). ERp57 is a protein disulfide isomerase (PDI) which mediates thiol-disulfide interchanges during post-translational folding of glycoproteins (8, 9). Structurally, both share common features that determine their primary ER-localization, such as an N-terminal signal peptide directing translational synthesis into the ER-lumen, and a C-terminal K/QDEL ER-retention motif. Increasingly, studies have identified extra-ER localization of either protein that has implicated additional non-chaperone related functions (10, 11). Within this context, the extra-ER roles of CRT have been more intensely studied, in part due to its deemed significance as an immunogen for professional phagocytes. However, ERp57 and CRT directly interact to form a stable complex (12, 13), thus it is likely that ERp57 would be present in the same extra-ER locales as CRT.

The integrin family of transmembrane cell adhesion receptors are heterodimers composed of an α- and a β- subunit (14). Integrin activation describes a functional switch from a low- to a high-affinity ligand binding state, with accompanying conformational changes to the extracellular, transmembrane and cytoplasmic domains of the αβ-subunits (15). We, and others, have shown that cell adhesion enhances α-integrin interaction with CRT, in a manner dependent on the cytosolic GFFKR peptide motif (16–18). The human genome encodes up to 21 α-integrin isoforms with sequence-unique cytoplasmic domains except for the conserved juxtamembrane GFFKR motif that forms the inner membrane clasp with its β-subunit counterpart (14, 19). Since protein interactions involving integrin cytoplasmic tails form the basis for integrin adhesion-mediated signaling, the GFFKR-mediated integrin-CRT interaction may constitute a conserved signaling mechanism attributable to the α-subunit for any given αβ-heterodimer.

Previously, we showed that leukemic blasts treated with ICD-inducers while engaging integrin substrates exhibited reduced presentation of cell surface CRT, suggesting that integrin function can suppress ICD (20). We characterized the successful release of CRT from the ER to the cytosol, but which failed to present on the surface of cells with activated integrins. This phenomenon can be explained as CRT being sequestered within the cytosolic space as a result of binding with the juxtamembrane GFFKR motif of α-integrins. In the present study, we assessed both the roles and surface presentation characteristics of ERp57 and CRT in coordination with integrin function. Our findings indicate that ERp57, CRT, and α-integrins, via their mutual pairwise interactions, coordinate to regulate surface CRT presentation in ICD.

## Materials and Methods

### Human T-ALL Cell Lines and Cells

Jurkat cells were obtained from ATCC. The Jurkat-derivatives Tacδ and Tacδ^scr^ were described previously (16). Dr. Andrew Weng (University of British Columbia) provided REX, THP-6, SUP-T1, and DND-41 T-ALL cells. Cells were cultured at 37°C, 5%CO_2_ in complete RPMI (cRPMI is RPMI-1640, 10% FBS, pen-strep and nonessential amino acids [ThermoFisher]). Cell transfection was done by nucleofection (Lonza).

T-ALL primary sample from diagnosis and samples from subsequent relapses of the same patient were obtained from the BC Children's Hospital Biobank with ethics approval from the BC Women's and Children's Hospital institutional review board (H12-03216). Patient bone marrow aspirate was injected via tail vein into NOD-SCID/IL-2Rγ null **(**NSG) mice (Jackson Laboratory). Mice were monitored for human leukemia engraftment by flow cytometry analysis of peripheral blood. Those with high leukemia burden were euthanized and their spleens (~80% human CD45^+^ lymphoblasts) immediately sourced for human T-ALL cells.

### CRISPR-Cas9 Generation of CRT^−/−^, ERp57^−/−^, and β1^−/−^ Jurkat Cell Lines

CRISPR-Cas9 generation of CRT^−/−^ cells was described previously (20). ERp57^−/−^ cells were generated using 5′CCGACGTGCTAGAACTCACG3′ as guide DNA targeting *PDIA3* exon1. β1^−/−^ cells were generated using 5′TTTGTGCACCACCCACAATT3′ as guide DNA targeting *ITGB1* exon2. Guides were cloned into plasmid pX458 (21), transfected by nucleofection (Amaxa, Lonza) into WT Jurkat cells, and 24 h later flow-sorted for Cas9-GFP positive single cells to isolate transfected clones. ERp57^−/−^ clones were identified by Western blotting, while β1^−/−^ clones were identified by flow cytometry for loss of the respective protein expression. PCR amplicons for the targeted genomic loci for each clone was sequenced to confirm indel formation. The data shown in this manuscript is for the representative clones Erp57-1.1 (ERp57^−/−^) and JCb1-2.8 (β1^−/−^), with the major phenotypes reproduced in at least one other independently derived clone.

### Flow Cytometry

LSRFortessa™ and Accuri™ C6 were used for analytical work and FACSAria™ (BD) for cell sorting. Post-acquisition analysis was done using FlowJo (Tree Star).

### Antibodies

Antibodies for flow cytometric detection of surface antigens: α2-integrin (P1E6-C5), α3-integrin (ASC-1), α4-integrin (9F10), α5-integrin (NKI-SAM-1), α6-integrin (GoH3), F4/80 (BM8, BioLegend); β1-integrin (sc-53711, Santa Cruz); CRT (ab2907), ERp57 (ab10287, Abcam). Antibodies for flow cytometric detection of intracellular antigens: CRT (ab2907), PDI (ab2792) and cytochrome C (6H2.B4, Biolegend). Antibodies for immunoprecipitation and immunoblotting: α4-integrin (HP2/1), CRT (PA3-900, ThermoFisher), ERp57 (ab10287, Abcam), and GAPDH (FF26A/F9, BioLegend). Antibodies for integrin activation and phagocytosis assays: β1-integrin (9EG7) and CD47 (B6H12, BD Biosciences).

### Cell Surface Calreticulin and ERp57 Assays

Cells were serum-starved in blank RPMI (bRPMI) for 12 h before experiments. Cells were resuspended in bRPMI at 10^6^ cells/mL and treated with 4 μg/mL doxorubicin (Sigma-Aldrich) for 4 h or 300 μM oxaliplatin (Tocris) for 2 h, at 37°C. Oxaliplatin is the preferred ICD-inducer used in certain assays to mitigate the fluorescence of doxorubicin. For adhesion assays, cells were seeded on fibronectin or bovine serum albumin (BSA) coated wells 1 h before drug treatments. In some experiments, cells were treated with 1 μg/mL integrin antibodies at 15 min prior to and during the drug treatment. Some cells received 1 mM MnCl_2_ during the last 30 min of drug treatment. Antibody-based flow cytometry was used to assess surface CRT or surface ERp57 levels using 633 nm excitation of secondary conjugates. Geometric Mean Fluorescence Intensity (gMFI) calculation was assessed for non-apoptotic gated cells (Annexin V –ve).

### Cell Permeabilization and Immuno-Staining for CRT and ERp57

Following treatments, cells were fixed in 3.7% formaldehyde/PBS for 15 min and divided into 2 groups, each treated with 0.1% Triton X-100/PBS for 5 min or with 25 μg/mL digitonin/PBS (Acros) for 5 min. Following PBS washes, cells were immuno-stained for CRT and ERp57 before analysis using flow cytometry. We optimized digitonin concentrations based on the ability to maintain ER integrity that precluded staining for the ER-marker, PDI (20).

### Immunoprecipitation and Immunoblot Analysis

Lysates were prepared in PN buffer [10 mM PIPES, 50 mM NaCl, 150 mM sucrose, 50 mM NaF, 40 mM Na_4_P_2_O_7_.10H_2_O, 1 mM CaCl_2_, 1 mM MgCl_2_, 1%Triton X-100, Complete protease inhibitors (Roche)]. For α4 immunoprecipitation, 1 mg lysate was incubated with 1 μg HP2/1 antibody and precipitated with protein A/G-Sepharose (Pierce).

### Phagocytosis Assay

Phagocytosis assays were conducted as described previously (20, 22). Briefly, femura and tibiae bone marrow aspirates of 8-week-old C57BL/6 mice were plated at 5 × 10^5^ cells/mL for 4 h in complete IMDM (cIMDM, 10% FBS, pen/strep). Non-adherent cells were re-plated in cIMDM with 10 ng/mL murine MCSF (StemCell) for 10 days. Adherent cells (macrophages) from 10 day cultures were >95% Mac-1^+^ and F4/80^+^. Macrophages were lifted and starved for 1 h in IMDM before co-incubating with target cells.

Target Jurkat cells were pre-labeled with CellTracker (Invitrogen) per manufacturer's instructions. Cells were co-incubated for 2 h with 7 μg/mL α-CD47 (B6H12) and/or 300 μM oxaliplatin. In some experiments, cells were also co-incubated with 1 μg/mL 9EG7 antibody. Phagocytosis was initiated by co-plating 2 × 10^5^ macrophages with 1 × 10^6^ washed Jurkat, CRT^−/−^ or ERp57^−/−^ cells in low adhesion 24-well plates (ThermoFisher) for 2 h in IMDM at 37°C. Macrophages were stained with F4/80 antibodies, and total cell mixture analyzed by flow cytometry. Phagocytosis calculation: % Phagocytosis = 100^*^(CellTracker^+^, F4/80^+^ macrophages/total macrophages).

### Statistical Analysis

Statistical analysis was performed using GraphPad Prism. Error bars are the standard deviation values obtained from at least three treatment replicates conducted within an experiment. All data shown are representative of 2–3 independently conducted experiments. *P*-values were calculated using two-way ANOVA with Tukey's multiple comparisons test.

## Results

### Requirement of CRT or ERp57 for ICD-Induced Surface Presentation

**Figure 1:**
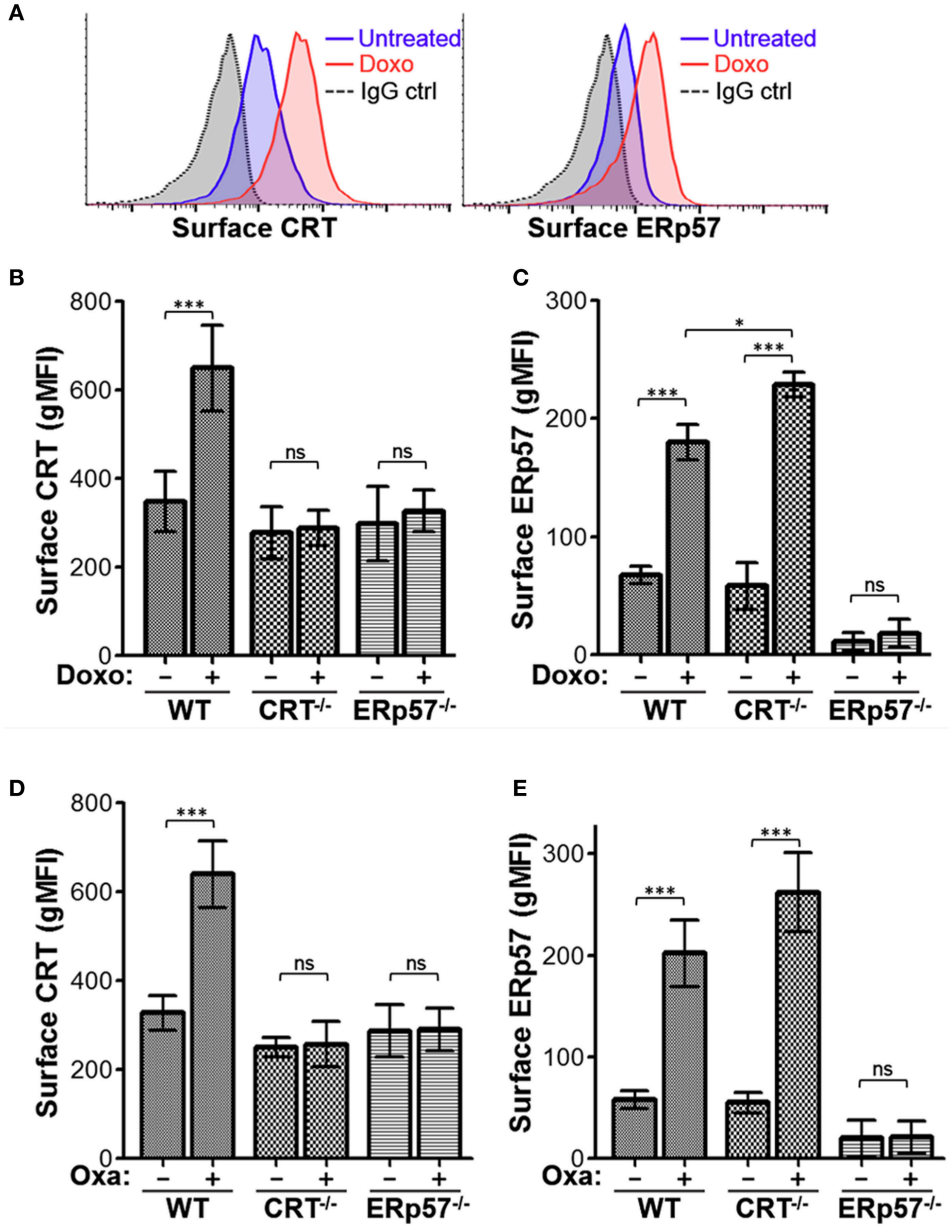
ERp57 is presented on the surface of lymphoblasts treated with ICD-inducers and required for the surface presentation of CRT. **(A)** Representative flow cytometry plots of Jurkat wildtype (WT) cells treated with or without doxorubicin (Doxo) and stained for surface CRT or ERp57. **(B,C)** Flow cytometry gMFI plots of surface CRT and surface ERp57 for WT, CRT^−/−^, and ERp57^−/−^ cells treated with or without doxorubicin. **(D,E)** Similar experiment for cells treated with or without oxaliplatin (Oxa). Plotted are the mean ± S.D.; *n* = 3; *p*-values: *** < 0.001; * < 0.05; ns, not significant. Data shown are representative of three independently conducted experiments.

Previously, we derived CRT^−/−^ Jurkat cells using CRISPR-Cas9 technology and observed that doxorubicin treatment of CRT^−/−^ cells resulted in increased cell surface ERp57, indicating that CRT is not required for surface translocation of ERp57 (20). To assess the requirement for ERp57 in CRT translocation in the same cell model, we used CRISPR-Cas9 to generate indel mutations within the first coding exon of *PDIA3* (Supplementary Figure 1A). Immunoblot analysis confirmed loss of ERp57 protein expression, and reduced total CRT expression (Supplementary Figure 1B). As before (20), WT cells treated with the ICD-inducing agent, doxorubicin, exhibited increased cell surface levels of CRT and ERp57 as assayed by flow cytometry (Figure 1A). Next, we compared surface CRT and surface ERp57 for WT, CRT^−/−^, and ERp57^−/−^ cells stimulated with either doxorubicin (Figures 1B,C) or oxaliplatin (Figures 1D,E) as ICD-inducers. Surface CRT is abolished in CRT^−/−^ and ERp57^−/−^ cells with treatment, indicating that ERp57 is essential for surface translocation of CRT (Figures 1B,D). Herein we confirm that surface ERp57 is increased in ICD-induced WT and CRT^−/−^ cells, consistent with our previous observation that CRT is not required for surface ERp57 translocation. We also observed that ICD-induced surface ERp57 levels are significantly higher for CRT^−/−^ compared to WT cells (Figures 1C,E), even though total ERp57 levels are comparable (Supplementary Figure 1B). This raised the intriguing possibility that CRT may have a role to reduce the cell surface presentation of ERp57.

### Cells Lacking CRT or ERp57 Exhibit Reduced Macrophage-Mediated Phagocytosis

**Figure 2:**
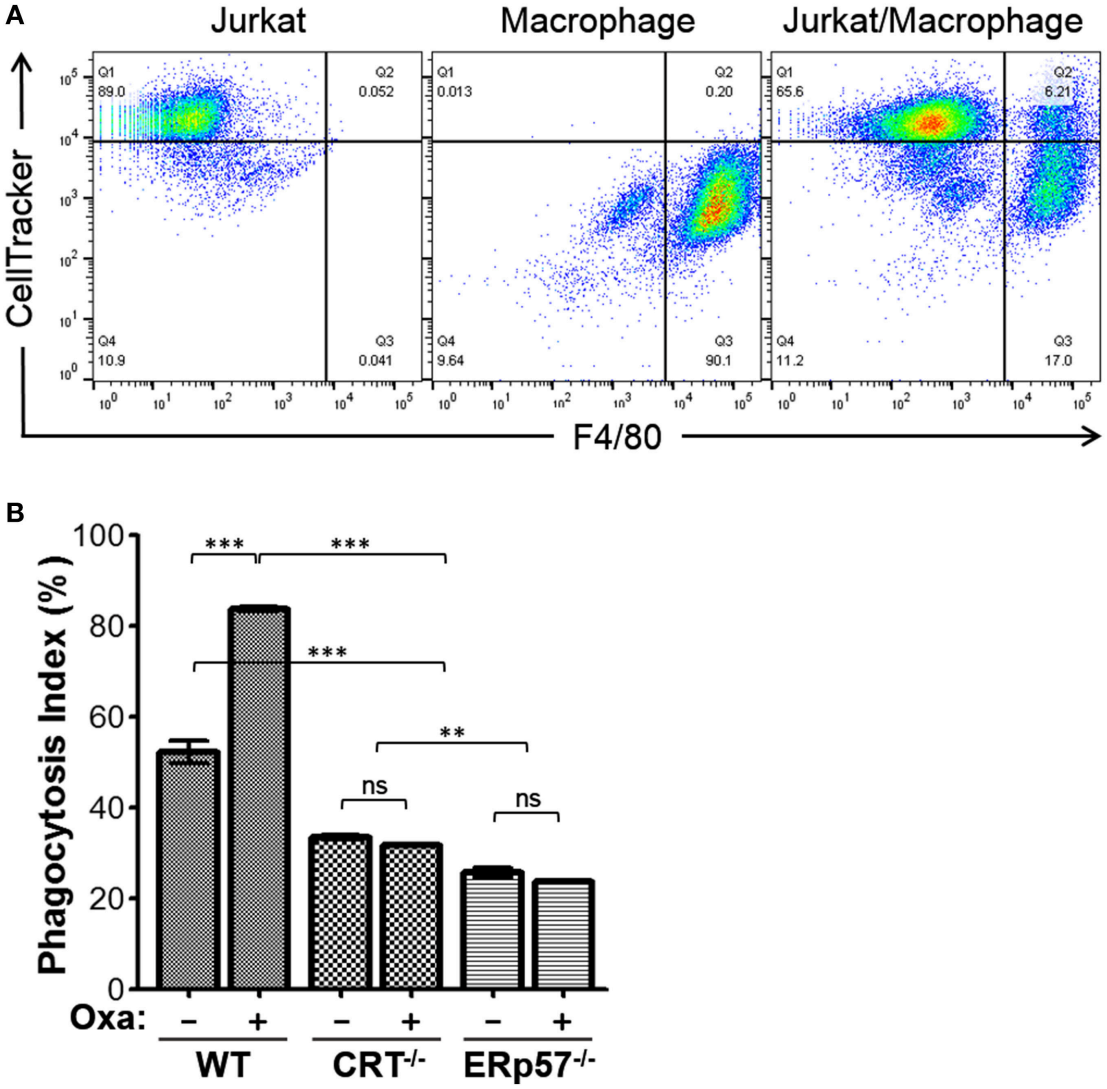
Phagocytosis assay for WT, CRT^−/−^, and ERp57^−/−^ cells. CellTracker-labeled Jurkat WT, CRT^−/−^, or ERp57^−/−^ cells were untreated or treated with oxaliplatin, followed by co-incubation with F4/80-labeled primary mouse macrophages. **(A)** Representative flow cytometry plots showing input target (Jurkat) and/or effector macrophages. The dual positive gated events are indicative of phagocytosis. **(B)** The calculated phagocytosis index depicted as bar graphs for the indicated target cells and treatments. Plotted are the mean ± S.D.; *n* = 3; *p*-values: *** < 0.001; ** < 0.01; ns, not significant. Data shown are representative of at least three independently conducted experiments.

Since ERp57^−/−^ cells show no increased presentation of surface CRT upon drug treatment, we would expect reduced target cell engulfment by professional phagocytes. To evaluate this, we performed a phagocytosis assay using murine bone marrow-derived macrophages as effector cells, and the Jurkat-derivative WT, CRT^−/−^, and ERp57^−/−^ as target cells. Target cells prelabeled with CellTracker were treated with or without oxaliplatin, followed by co-incubation with macrophages, and the labeled cell mixture analyzed by flow cytometry to delineate the various populations. To facilitate phagocytosis, the anti-phagocytic CD47 signal on all target cells is also negated by treatment with the function blocking antibody, B6H12 (20, 22). CellTracker and F4/80 double positive gated events are indicative of phagocytosis (Figure 2A) and the calculated phagocytosis index for all conditions is shown in Figure 2B. We found that phagocytosis was significantly increased for WT cells with oxaliplatin treatment, while oxaliplatin treatment of CRT^−/−^, or ERp57^−/−^ cells failed to promote phagocytosis. Taken together, our results show that loss of surface CRT due to loss of ERp57 effectively reduced phagocytosis by macrophages to a degree equivalent to cells lacking endogenous CRT. These data confirmed the importance of ERp57 as a facilitator of ICD, acting to present CRT on the cell surface as an “eat me” immunogen.

### Accumulation of Cytosolic CRT Is Dependent on ERp57, but Not Vice Versa

**Figure 3:**
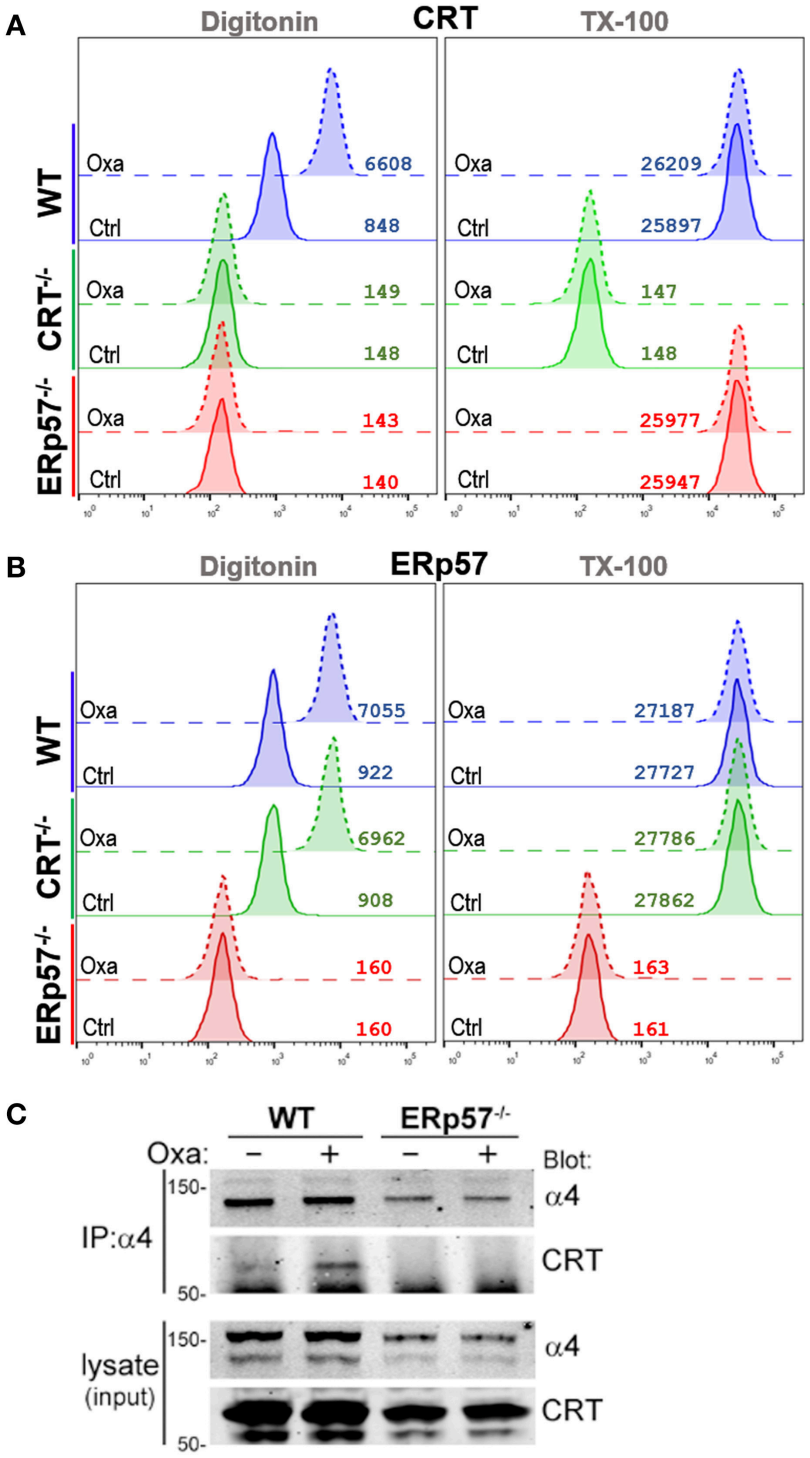
Drug-induced ICD promotes cytosolic accumulation of CRT and ERp57. **(A,B)** WT, CRT^−/−^, and ERp57^−/−^ cells were untreated (Ctrl) or treated with oxaliplatin (Oxa), fixed in suspension and either permeabilized with Digitonin or Triton X-100, and stained for CRT or ERp57, as indicated. Plotted are flow histograms for an experiment conducted in triplicates, and representative of at least two independent experiments. Numbers are the computed mean fluorescence intensity (MFI). **(C)** Integrin-α4 was immunoprecipitated (IP) from lysates of non-treated or oxaliplatin-treated WT and ERp57^−/−^ cells. As shown are immunoblots of integrin-α4 and CRT. Representative of at least two independently conducted experiments.

To gain insight on the interdependence of ERp57 and CRT trafficking in ICD, we assessed their accumulation in the cytosolic, extra-organellar compartment following ICD-induction using flow-based detection of intracellular proteins for differentially permeabilized cells (20). Oxaliplatin-treated WT cells that were partially permeabilized with Digitonin show detectable increases in CRT, but not of the ER-protein PDI or mitochondrial-protein cytochrome C (Supplementary Figure 2). This increase was not observed for cells fully permeabilized with TX-100, suggesting that Digitonin achieved plasma membrane permeabilization but not of the ER nor mitochondrial compartments (Figure 3). We found that oxaliplatin treatment promoted the increases of cytosolic CRT in WT cells, but not in ERp57^−/−^ cells (Figure 3A), suggesting that the extra-ER accumulation of CRT is ERp57-dependent. In contrast, oxaliplatin treatment promoted cytosolic increases of ERp57 in WT and CRT^−/−^ cells (Figure 3B), suggesting that the extra-ER accumulation of ERp57 is CRT-independent. This result mirrors that observed for surface ERp57 and CRT (Figure 1), consistent with an interpretation that ICD-induced translocation of CRT from ER to cytosol and then to the cell surface being critically dependent on ERp57 as a co-trafficking protein.

It's been shown that CRT can interact with the cytosolic GFFKR motif of α-integrins (18, 23, 24). Oxaliplatin treatment of WT cells resulted in increased levels of CRT that co-immunoprecipitated with α4-integrins (Figure 3C). In contrast, CRT was not detected in α4-immunoprecipitates of ERp57^−/−^ cells, indicating that the α4-CRT interaction occurs within the cytosol, but is facilitated by ERp57 promoting the movement of CRT out of the ER.

### Integrin Cell Adhesion Modulates Surface CRT and ERp57

**Figure 4:**
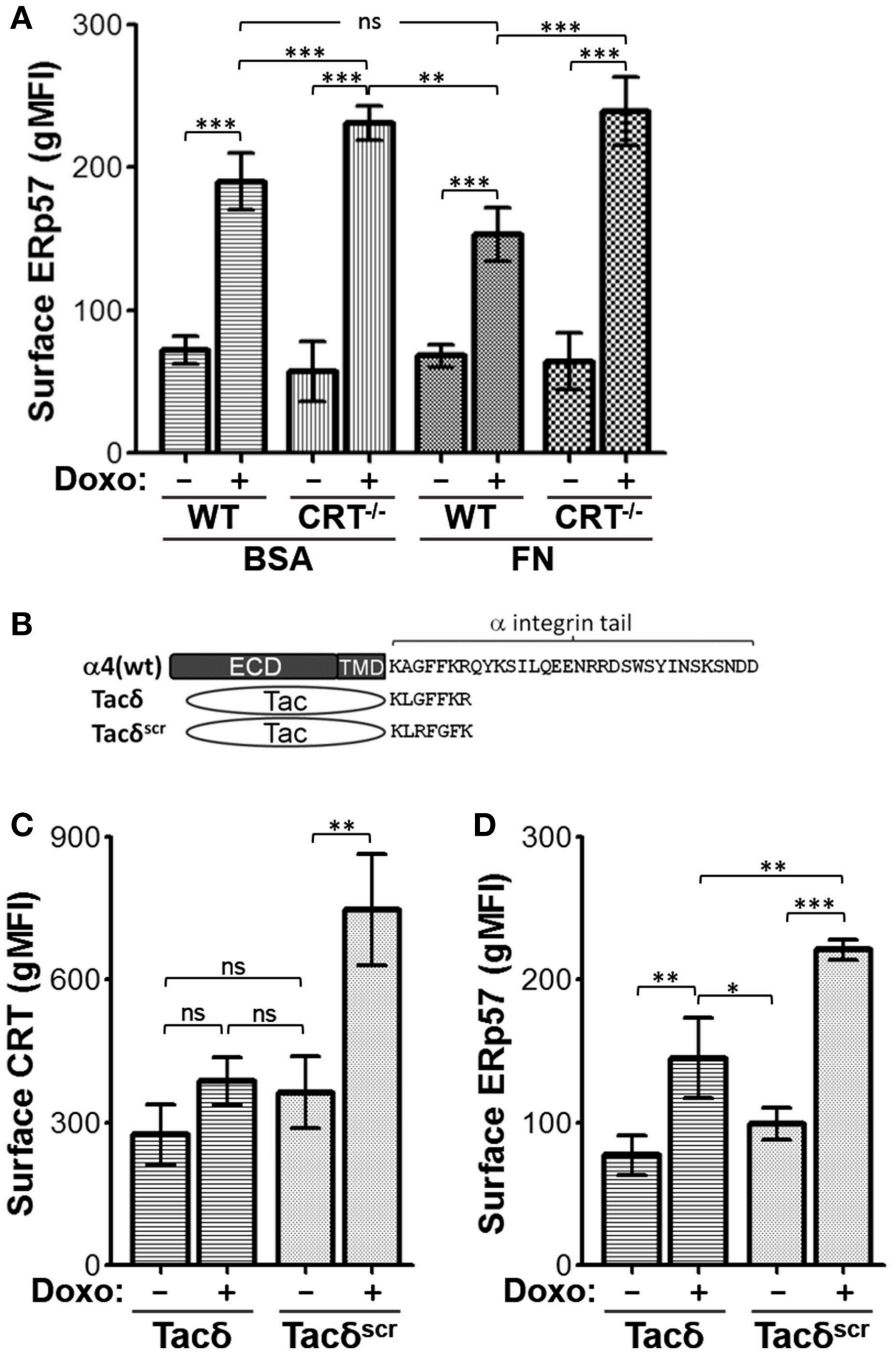
Integrin function modulates ICD-induced surface CRT and ERp57. **(A)** WT and CRT^−/−^ cells pre-seeded on fibronectin (FN) or BSA substrates were treated with or without doxorubicin and surface ERp57 determined by flow cytometry. **(B)** Schematic of constructs used in relation to α4-integrin. ECD-extracellular domain; TMD-transmembrane domain. Tacδ and Tacδ^scr^ are fusions of ECD and TMD of Tac with the cytoplasmic peptide, KLGFFKR, or the scrambled version, KLRFGFK, respectively. **(C,D)** Jurkat cells expressing Tacδ or Tacδ^scr^ in suspension were treated with or without doxorubicin and surface CRT or ERp57 determined by flow cytometry. Flow gMFI plots **(A,C,D)** are the mean ± S.D.; *n* = 3; *p*-values: *** < 0.001; ** < 0.01; * < 0.05; ns, not significant. Data shown are representative of three independently conducted experiments.

Previously, we reported that integrin function led to reduced ICD, a phenomenon manifested by reduced CRT at the cell surface (20). Given that CRT and ERp57 can exist as a complex in the ER, and that ERp57 is required for CRT translocation to the cell surface, we assessed if integrin function may also modulate surface ERp57 levels (13, 25–27). Cells were plated on fibronectin (FN) to engage integrins, or on BSA as a control substrate, and treated with or without doxorubicin. When plated on BSA, surface ERp57 levels were lower in drug-treated WT cells compared to CRT^−/−^ cells (Figure 4A). This result is comparable to that obtained with cells treated in suspension (Figure 1). WT cells plated on FN resulted in surface ERp57 levels that was further reduced when compared to CRT^−/−^ cells on BSA or FN, although the difference was not significant when compared to WT cells on BSA (Figure 4A). Importantly, these results lend further support for a negative modulatory role for CRT in the surface translocation of ERp57.

CRT is known to bind the juxtamembrane cytosolic GFFKR peptide motif of α-integrins (16, 24). Previously, we showed that cells expressing Tacδ, a chimeric non-integrin receptor bearing GFFKR as the cytosolic domain, constitutively binds CRT and reduces drug-induced surface CRT (16, 20). We now show that Tacδ cells exhibit a similar reduction of surface CRT and ERp57 when compared to the control Tacδ^scr^ cells, which bears a scrambled RFGFK motif that does not bind CRT (Figures 4B–D). Importantly, the results indicate that the cytosolic GFFKR motif on Tacδ is sufficient for down modulating surface CRT and ERp57 in a constitutive manner that bypasses the requirement for cell adhesion as the inhibitory switch.

### Integrin Activation by Agonists Reduces Surface CRT and ERp57

**Figure 5:**
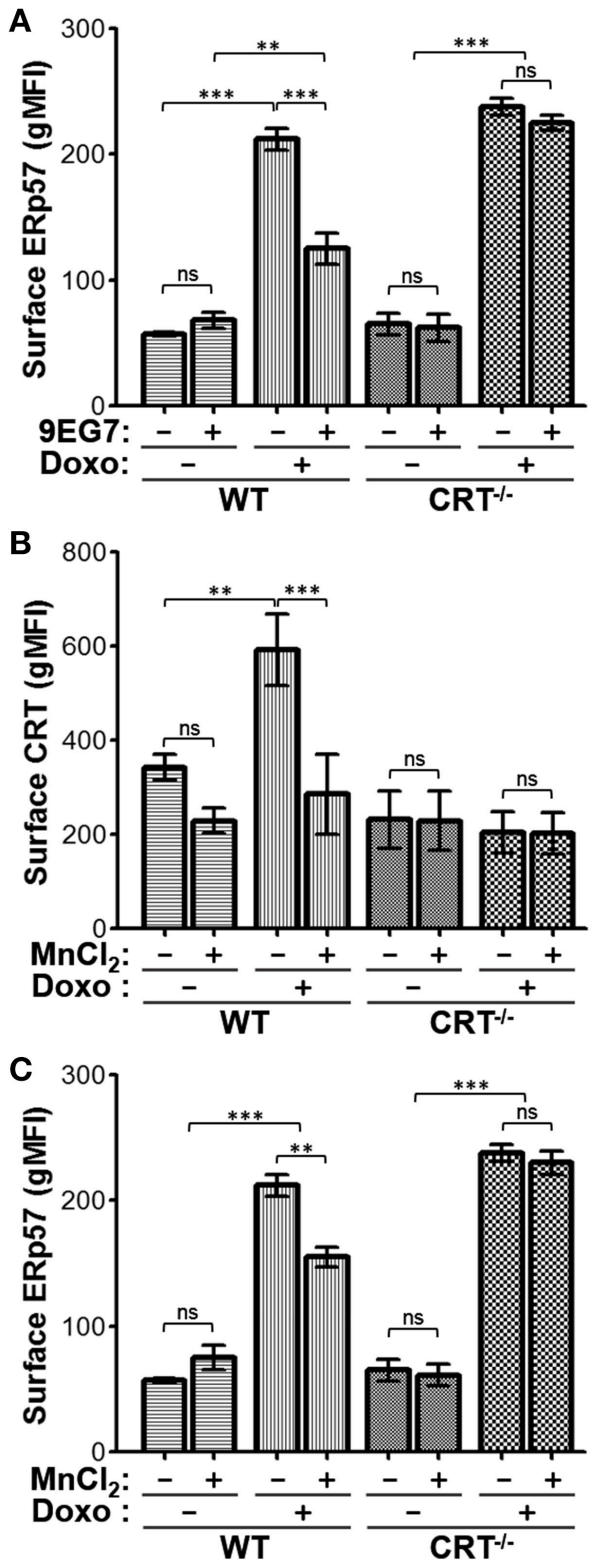
Jurkat cells treated with integrin-activating agonists exhibit reduced surface CRT and ERp57. **(A)** WT or CRT^−/−^ cells were treated with combinations of doxorubicin and/or 9EG7 mAb and surface ERp57 determined by flow cytometry. **(B,C)** WT and CRT^−/−^ cells were treated with combinations of doxorubicin and MnCl_2_ and surface CRT or surface ERp57 determined by flow. Flow gMFI plots are the mean ± S.D.; *n* = 3; *p*-values: *** < 0.001; ** < 0.01; ns, not significant. Data shown are representative of at least two independently conducted experiments.

The negative modulatory effect of integrins on drug-induced surface CRT can be further enhanced by treating cells with agonists which induce integrin activation. We had shown that cells pre-incubated with the β1-integrin activating antibody, 9EG7, led to complete inhibition of drug-induced surface CRT (20). We repeated this experiment to assess the effects on surface ERp57. As expected, surface ERp57 was reduced on WT cells treated with doxorubicin and 9EG7, when compared to the control IgG-treated group (Figure 5A). In contrast, surface ERp57 levels were not reduced upon 9EG7-treatment of CRT^−/−^ cells (Figure 5A). Our results using intracellular flow cytometry (Figures 3A,B) indicated that Digitonin-permeabilized cells exhibit increases in CRT and ERp57. To gain some insight on this phenomenon, we subjected WT cells to treatments with and without oxaliplatin and 9EG7, and imaged Digitonin-permeabilized cells following immunofluorescence labeling for CRT or ERp57 (Supplementary Figure 3). We observed similar increased staining for CRT or ERp57 in oxaliplatin-treated cells both with and without co-treatment with 9EG7, consistent with the interpretation that 9EG7-mediated reduction of surface CRT or ERp57 occurs independently of their presumptive release from the ER.

Further to the results seen with the antibody-based integrin agonist, we assessed if Mn^2+^-mediated activation of integrins would also reduce surface CRT and ERp57 presentation. Similar to 9EG7, Mn^2+^ reduced surface CRT levels in WT cells treated with doxorubicin (Figure 5B). To a lesser degree, Mn^2+^ also reduced surface ERp57 in doxorubicin-treated WT cells, while having no effect on CRT^−/−^ cells (Figure 5C). Thus, despite the fact that CRT is not required for surface translocation of ERp57, the presence of CRT appears to modulate the extent of this translocation. The negative modulatory effect on surface ERp57 resulting from activated integrins appears to be an indirect one, where cytosolic CRT-integrin interaction reduces ERp57-CRT translocation as a complex to the cell surface. In cells lacking CRT, this passenger effect is nullified, with ERp57 able to translocate freely and maximally in the presence of activated integrins (Figures 5A,C).

### Reduced Integrin Expression Increases Drug-Induced Surface CRT and ERp57

**Figure 6:**
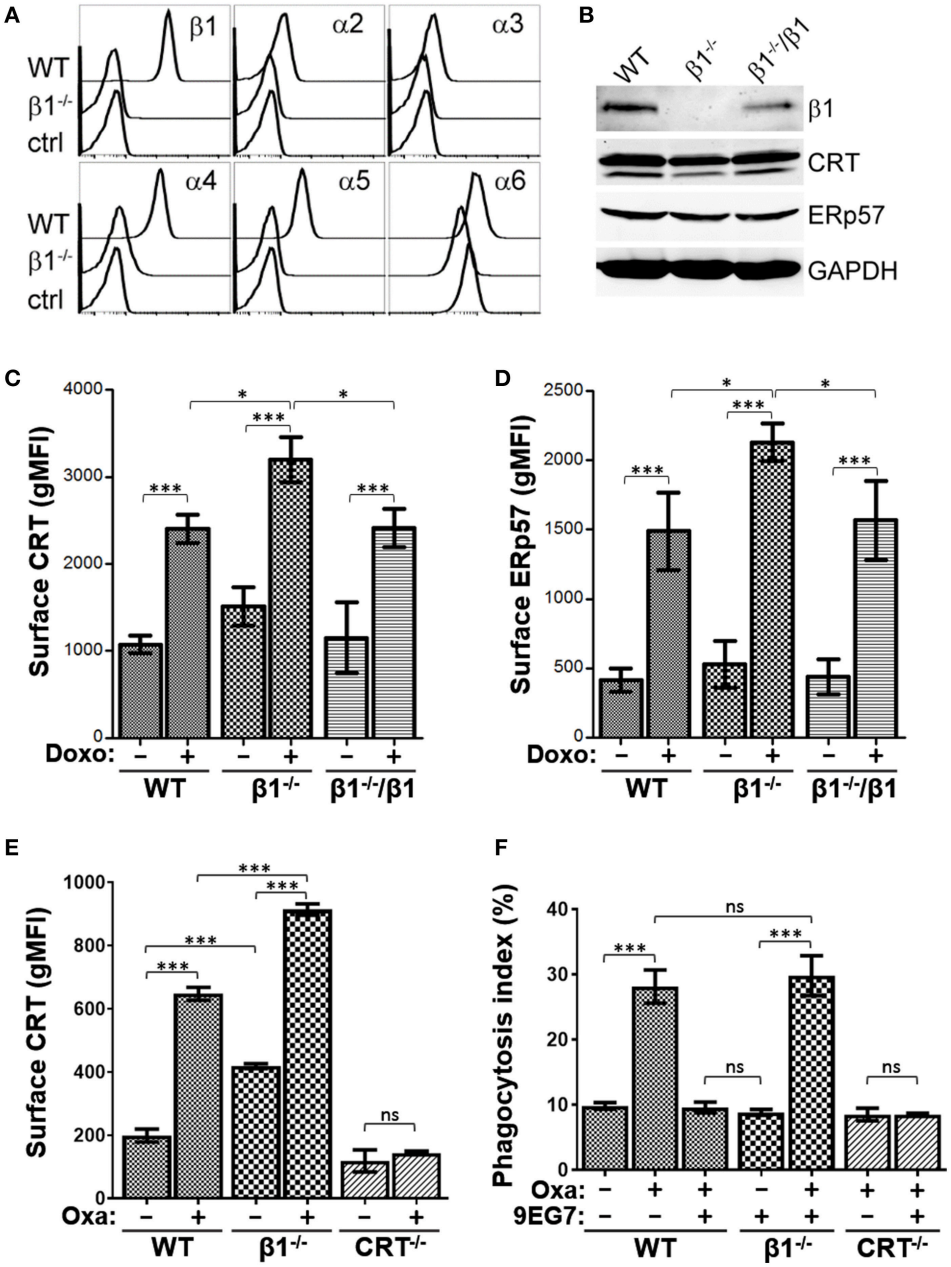
Reduced integrin expression increases surface CRT and ERp57. **(A)** Flow histograms for the indicated integrin expression in WT and β1^−/−^ cells. **(B)** Immunoblot assay for expression of integrin β1, CRT, ERp57, and GAPDH in lysates of WT, β1^−/−^, and β1^−/−^ cells re-expressing β1. **(C,D)** The indicated cells were treated with or without doxorubicin and surface CRT or ERp57 determined by flow cytometry. **(E)** WT, β1^−/−^, and CRT^−/−^ cells were treated with or without oxaliplatin and surface CRT determined by flow cytometry. **(F)** Phagocytosis assay for WT, β1^−/−^, and CRT^−/−^ cells treated with oxaliplatin or 9EG7 as indicated. Plots are the mean ± S.D.; *n* = 3; *p*-values: *** < 0.001; * < 0.05; ns, not significant.

Given that activated integrins have a negative effect on drug-induced surface CRT and ERp57 presentation, we reasoned that cells with reduced integrin expression may exhibit a corresponding increase in drug-induced ICD. Stable integrin expression requires the αβ-heterodimer, thus a most efficient way to reduce overall integrin expression is to abrogate the β1-subunit to obtain decreased expression of multiple α-integrins. We generated a β1^−/−^ clonal strain of Jurkat cells by CRISPR-Cas9 (Supplementary Figure 4A), and showed reduced expression of β1-paired α2, α3, α4, α5, and α6 integrins (Figure 6A). Expression of αV and β3 integrins were slightly elevated in β1^−/−^ cells, suggestive of compensatory expression, while all others assessed were unchanged (Supplementary Figure 4B). Total CRT and ERp57 expression were comparable between WT and β1^−/−^ cells (Figure 6B). As predicted, β1^−/−^ cells treated with doxorubicin exhibit significantly higher surface CRT and ERp57 levels compared to the parental WT cells or β1^−/−^ cells re-expressing β1-integrins (Figures 6C,D, Supplementary Figure 4C), consistent with the interpretation that reduced integrin expression enhances CRT exposure on the surface of tumor cells treated with ICD-inducers.

To assess if integrin modulation of tumor cells functionally modulates their ability to be phagocytosed by macrophages, we first confirmed that oxaliplatin-treated β1^−/−^ cells exhibit higher surface CRT when compared to WT cells (Figure 6E). As expected, 9EG7-treatment of WT cells effectively negated the increase in phagocytosis induced with oxaliplatin (Figure 6F). This neutralizing effect was not observed in β1^−/−^ cells, consistent with 9EG7 as a specific antibody to β1-integrin. The phagocytosis index for oxaliplatin-treated WT and β1^−/−^ cells were comparable, suggesting the increase in surface CRT seen in β1^−/−^ cells when compared to WT is not sufficient to alter their ability to be phagocytosed.

### Integrin Activation Modulates Surface CRT and ERp57 in T-ALL

**Figure 7:**
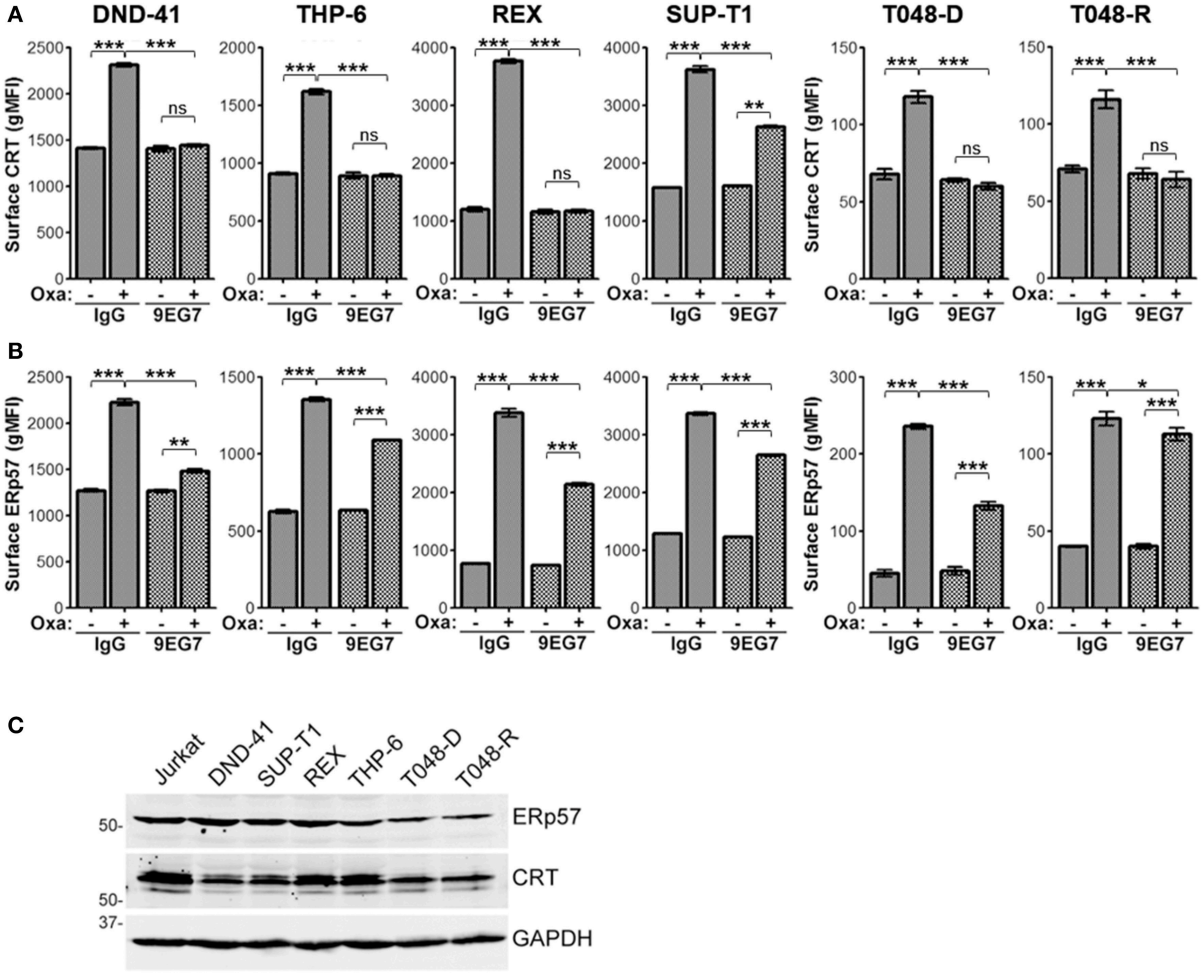
Treatment of various T-ALL lymphoblasts with 9EG7 and oxaliplatin reduces surface exposure of CRT and ERp57. Flow gMFI plots for surface CRT **(A)** and surface ERp57 **(B)** for T-lymphoblasts treated with combinations of oxaliplatin and/or 9EG7 integrin antibodies. DND-41, THP-6, REX, and SUP-T1 are T-ALL cell lines. T048-D and T048-R are PDX T-ALL samples from a single patient taken at diagnostic and relapsed disease. Plots are the mean ± S.D.; *n* = 3; *p*-values: *** < 0.001; ** < 0.01; * < 0.05. Data shown are representative of at least two independently conducted experiments. **(C)** Immunoblot assay for expression of CRT, ERp57, and GAPDH in lysates of the indicated cells.

To determine if the decrease of surface ERp57 and CRT by integrins is not limited to our observation in Jurkat T-lymphoblasts, we repeated key assays using the T-ALL cell lines DND-41, THP-6, REX, and SUP-T1, as well as 2 primary human T-ALLs that had been expanded as xenografts (PDX). Cells were treated with combinations of oxaliplatin and/or 9EG7 and surface CRT or ERp57 assessed (Figures 7A,B). In agreement with the Jurkat cell observations, oxaliplatin-induced surface CRT and ERp57 was significantly reduced for all 4 T-ALL cell lines when cells also received 9EG7 integrin antibodies. Interestingly, 9EG7-mediated suppression of surface ERp57 was more pronounced in diagnostic leukemia of patient T048 (PDX T048-D) compared to its relapsed counterpart (PDX T048-R), even though both samples exhibited comparable suppression of surface CRT. In general, 9EG7-mediated reduction in surface CRT was greater than that observed for surface ERp57. We assessed, but found no obvious correlation for these observations as a function of total CRT and ERp57 expression (Figure 7C).

## Discussion

This study sought to understand the overlapping contributions for two CRT interacting proteins, ERp57 originating from the ER and integrins within the plasma membrane, in surface CRT presentation as a DAMP for cells treated with ICD-inducers. Our results highlight the indispensable role of ERp57 for CRT trafficking from the stressed ER to eventual surface presentation, while integrins possess regulatory roles in suppressing the extent of surface CRT presentation. Within the context of surface CRT levels presented during ICD, we propose that integrin function is anti-immunogenic, while ERp57 serves a pro-immunogenic function.

Cell surface ERp57, in the absence of CRT, is not known to be immunogenic. However, endogenous expression of ERp57 remains essential for trafficking of endogenous CRT to the cell surface. We used CRISPR to knockout expression of CRT or ERp57 in Jurkat lymphoblasts, and confirmed that drug-induced surface CRT required endogenous ERp57, while ERp57 is ably presented at the surface in the absence of endogenous CRT. Further analysis with differentially permeabilized cells indicated that cytosolic CRT was absent and not drug-inducible in ERp57^−/−^ cells, while cytosolic ERp57 was present and drug-inducible in CRT^−/−^ cells. Thus, the extra-ER localization of CRT appears dependent on ER-resident ERp57. Previous reports using CRT^−/−^ mouse embryonic fibroblasts had indicated that CRT and ERp57 were co-dependently translocated as a complex to the cell surface (4, 5). While this apparent discrepancy may prove to be cell type dependent, it is clear that surface trafficking of CRT is ERp57-dependent. In the absence of CRT, surface translocation of ERp57 may involve another ERp57 complex protein, calnexin, since cell surface calnexin is a feature seen in immature thymocytes (28).

Besides its surface presentation during ICD, reports have implicated multifaceted roles for extra-ER localized ERp57 that include: on the sperm head for mediating sperm-egg fusion (29), as a receptor for vitamin D3 (30), in protein complexes with NF-κB (31), all trans retinoic receptor (32), or STAT3 (33, 34), and as secreted forms with ECM remodeling potential (35, 36). Importantly, the functional contribution of ERp57 within these diverse locales may stem from either the disulfide isomerase activity and/or its role for co-trafficking lectin co-chaperones. Proposed explanations for extra-ER detection of chaperones such as CRT and ERp57 have included proteolysis of the ER-retention sequence, formation of protein complexes or conformation changes that mask its recognition, and saturation of the ER-retention machinery (37–40). These hypotheses center around avoidance of detection by KDEL receptors located within the Golgi apparatus for protein retrieval (41). Through differential permeabilization experiments, we characterized increased cytosolic CRT and ERp57 that originated from the ER for cells treated with ICD-inducers, while non-treated ERp57^−/−^ cells appear to be devoid of cytosolic CRT. Furthermore, cytosolic ERp57 can be detected in non-treated CRT^−/−^ cells, suggesting K/QDEL containing chaperones may exhibit differential retention potential mediated by the KDEL receptors. Given that CRT will remain ER-localized in the absence of ERp57, it is compelling to regard CRT-ERp57 as a sufficient and necessary “immunogenic complex” able to translocate to the cell surface.

In this study, we adopted several strategies to demonstrate the potential of α-integrins to inhibit the presentation of cell surface CRT. We show that engagement of integrins via adhesion, or enforced activation of integrins with an antibody or divalent cation agonists, either attenuated or severely suppressed ICD-induced surface CRT levels. Expression of a non-integrin chimeric receptor with α-integrin GFFKR as the cytosolic motif also led to low surface CRT levels. Given the highly conserved nature of GFFKR within the cytoplasmic domain of all known α-integrins, it is tempting to speculate that functional activation of any or all integrins could result in decreased surface CRT presentation and corresponding reduction in ICD. The transmembrane and cytoplasmic conformations of inactive and activated integrins have been studied in detail (15, 42, 43), facilitating a proposed mechanism whereby both the spatial separation of the α- and β- subunits, combined with tail “snorkeling” movements into the cytosolic space, allow for greater GFFKR exposure for activated α-integrins to bind cytosolic CRT. This may sequester CRT within the cytosol, reducing their levels presented on the cell surface during ICD.

We noted that surface ERp57 and CRT levels were correspondingly reduced for cells treated with integrin activating agonists. The reduced surface ERp57 observed is likely a passenger effect resulting from integrin binding to CRT of the CRT-ERp57 complex. This is consistent with the increased surface ERp57 seen in drug-treated CRT^−/−^ cells, where the interaction component for integrins is not expressed. Even though our study highlighted integrin's suppressive role for surface CRT presentation, it is within reason to speculate that integrin-mediated suppression of surface ERp57 may also reduce ERp57-specific functions at the cell surface.

It is important to note that the results of our study is limited to the use of CRT as a cell surface marker for induction of ICD in human lymphoblasts *in vitro*, coupled with exogenous manipulation of integrin function. As was proposed by the Nomenclature Committee on Cell Death, ICD should be defined as a form of regulated cell death sufficient to activate an adaptive immune response in immuno-competent hosts (44). The current accepted gold standard for demonstration of an ICD phenomenon is to model it *in vivo* using immuno-competent mice, with syngeneic tumor cells (45). Thus, a complete demonstration of functional integrin-mediated suppression of ICD *in vivo* will require a syngeneic murine model for ALL that is similarly manipulable with integrin agonists (e.g., 9EG7 activating antibodies) or genetically modified to express gain- or loss-of-function integrins.

Leukemia-stroma interactions involving integrins are known to confer pro-survival benefits to the tumor cells that include enhanced drug resistance, anti-apoptotic, and proliferative signaling. To that end, increased expression of particular integrins has been evaluated as negative prognostic markers for treatment response and outcomes (46). Our work adds to the plethora of integrins' pro-tumor functions that now include suppression of a surface immunogen important in innate anti-tumor immunity. Importantly, the conservation of the GFFKR-CRT interaction implies that all integrin-substrate interactions have the potential to suppress the anti-tumor effects of ICD. In addition, high levels of total integrin expression in any given tumor may also be predictive of reduced ICD response. Biologic therapeutics designed to target integrin-substrate interactions should be appropriately evaluated for oncology applications, since agents that block the tumor-stroma interaction by mimicking the substrate may inevitably activate integrins and reduce the ICD-response.

## Ethics Statement

This study was carried out in accordance with the recommendations of BC Women's and Children's Hospital institutional review board (REB) with written informed consent from all subjects. All subjects gave written informed consent in accordance with the Declaration of Helsinki. The protocol was approved by the BC Women's and Children's Hospital institutional review board (REB).

## Author Contributions

C-CL and CL designed research, analyzed data and wrote the paper. C-CL performed research. PL constructed β1^−/−^ cells. FP performed flow cytometry of β1^−/−^ cells. HV performed genomic sequencing for ERp57^−/−^ cells. MM and LS provided murine-derived macrophages. GR provided murine-expanded patient leukemia samples.

### Conflict of Interest Statement

The authors declare that the research was conducted in the absence of any commercial or financial relationships that could be construed as a potential conflict of interest.

## Supplementary Material

Supplemental Figure 1 CRISPR-Cas9 generation of ERp57^−/−^ Jurkat cells. **(A)** Sequencing of the *PDIA3* genomic loci showing single nucleotide insertion (red arrow) occurring at 77 bp from the predicted start codon and 5 bp from the PAM recognition motif (green bar). The frame shift mutated variant encodes for a predicted 65 amino acid protein product due to a premature termination codon. Wildtype ERp57 is 505 amino acids long. **(B)** Immunoblot analysis of Jurkat WT, ERp57^−/−^, and CRT^−/−^ cell lysates for expression of ERp57, CRT, and GAPDH.

Supplemental Figure 2 Drug-induced ICD promotes cytosolic accumulation of CRT, but not of PDI or cytochrome C. WT cells were untreated (Ctrl) or treated with oxaliplatin (Oxa), fixed in suspension and either permeabilized with Digitonin or Triton X-100, and stained for CRT, PDI, or cytochrome C, as indicated. Plotted are flow histograms for an experiment conducted in duplicates. Numbers are the computed mean fluorescence intensity (MFI).

Supplemental Figure 3 Immunofluorescence imaging of CRT and ERp57 in oxaliplatin treated WT cells. WT cells were untreated or treated with oxaliplatin (Oxa) or 9EG7, fixed in suspension, permeabilized with Digitonin, and stained for **(A)** CRT and **(B)** ERp57. Cells were mounted as a suspension in Prolong Gold with DAPI, and imaged at 0.3 μm slice intervals. As shown are representative Z-projection images of cells for each condition, green is CRT or ERp57, blue is DAPI-stained nucleus. Bar: 10 μm.

Supplemental Figure 4 CRISPR-Cas9 generation of integrin β1^−/−^ Jurkat cells. **(A)** Sequencing of the *ITGB1* genomic loci showing single nucleotide insertion (red arrow) in one allele and a 10 bp deletion (lower inset) in the other allele. PAM recognition motif indicated with green bar. Both frame shifted alleles encode for predicted truncated proteins of 64 and 65 amino acids resulting from premature termination codons. Wildtype β1 is 798 amino acids long. **(B)** Flow histograms for the indicated integrin expression in WT and β1^−/−^ cells. **(C)** Flow histogram of WT, β1^−/−^, and β1^−/−^/β1 cells for integrin β1 expression.
