# Supplementary figures and images for: Integrins and ERp57 Coordinate to Regulate Cell Surface Calreticulin in Immunogenic Cell Death
Source: Front Oncol. 2019 May 28;9:411. doi: 10.3389/fonc.2019.00411 (PMC6546883; doi:10.3389/fonc.2019.00411)

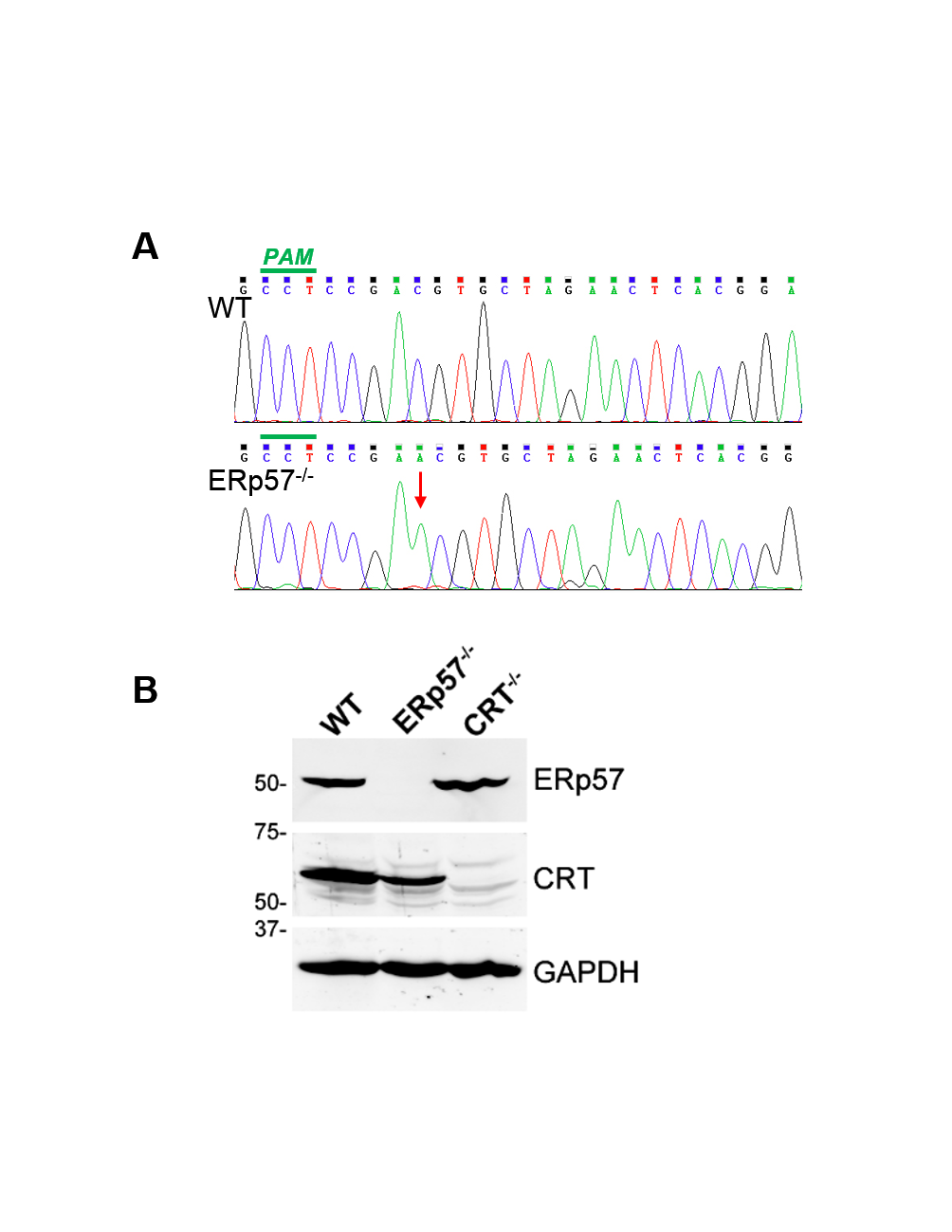

Supplement: Supplemental Figure 1 — CRISPR-Cas9 generation of ERp57−/− Jurkat cells. (A) Sequencing of the PDIA3 genomic loci showing single nucleotide insertion (red arrow) occurring at 77 bp from the predicted start codon and 5 bp from the PAM recognition motif (green bar). The frame shift mutated variant encodes for a predicted 65 amino acid protein product due to a premature termination codon. Wildtype ERp57 is 505 amino acids long. (B) Immunoblot analysis of Jurkat WT, ERp57−/−, and CRT−/− cell lysates for expression of ERp57, CRT, and GAPDH. [file Image_1.TIF]

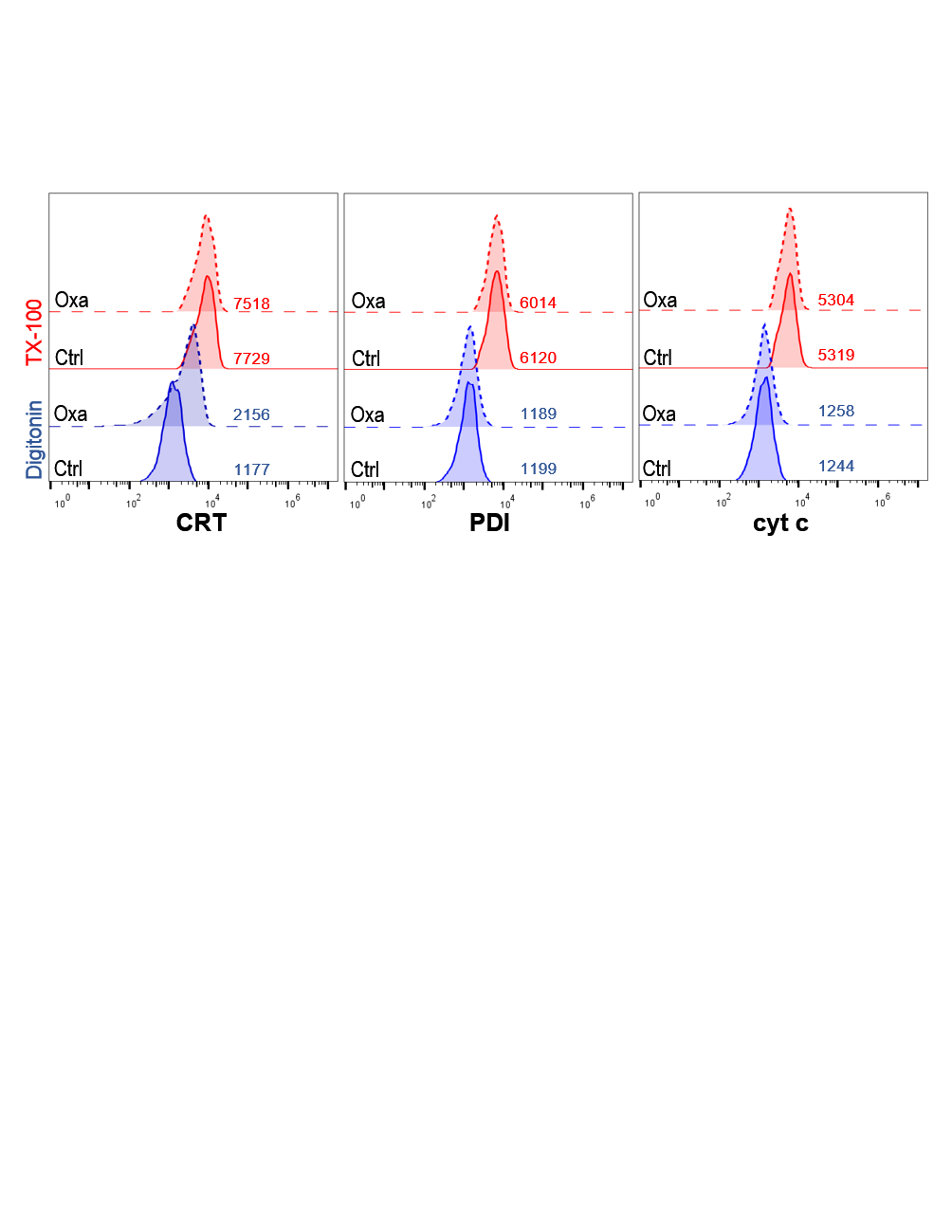

Supplement: Supplemental Figure 2 — Drug-induced ICD promotes cytosolic accumulation of CRT, but not of PDI or cytochrome C. WT cells were untreated (Ctrl) or treated with oxaliplatin (Oxa), fixed in suspension and either permeabilized with Digitonin or Triton X-100, and stained for CRT, PDI, or cytochrome C, as indicated. Plotted are flow histograms for an experiment conducted in duplicates. Numbers are the computed mean fluorescence intensity (MFI). [file Image_2.TIF]

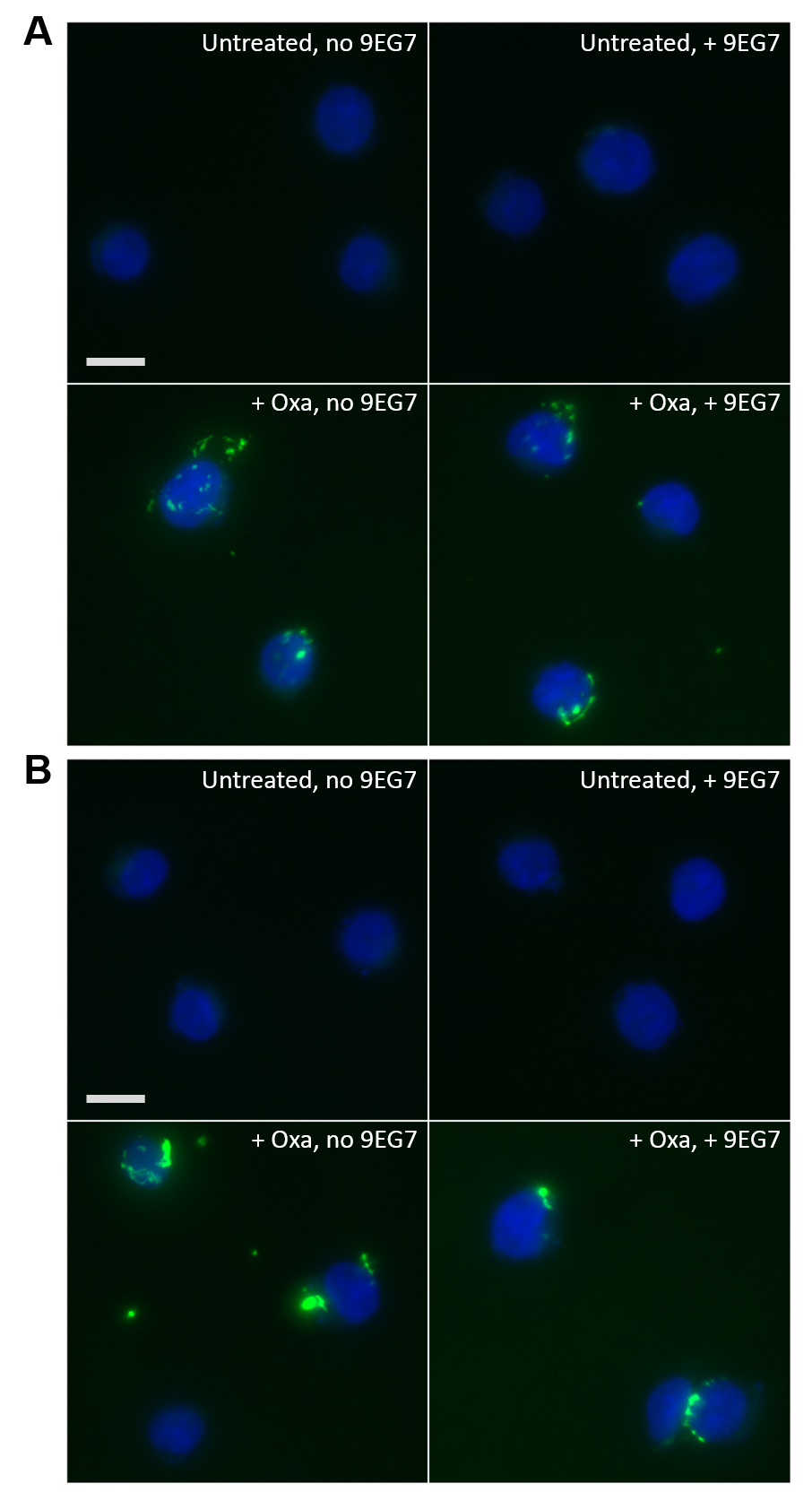

Supplement: Supplemental Figure 3 — Immunofluorescence imaging of CRT and ERp57 in oxaliplatin treated WT cells. WT cells were untreated or treated with oxaliplatin (Oxa) or 9EG7, fixed in suspension, permeabilized with Digitonin, and stained for (A) CRT and (B) ERp57. Cells were mounted as a suspension in Prolong Gold with DAPI, and imaged at 0.3 μm slice intervals. As shown are representative Z-projection images of cells for each condition, green is CRT or ERp57, blue is DAPI-stained nucleus. Bar: 10 μm. [file Image_3.TIF]

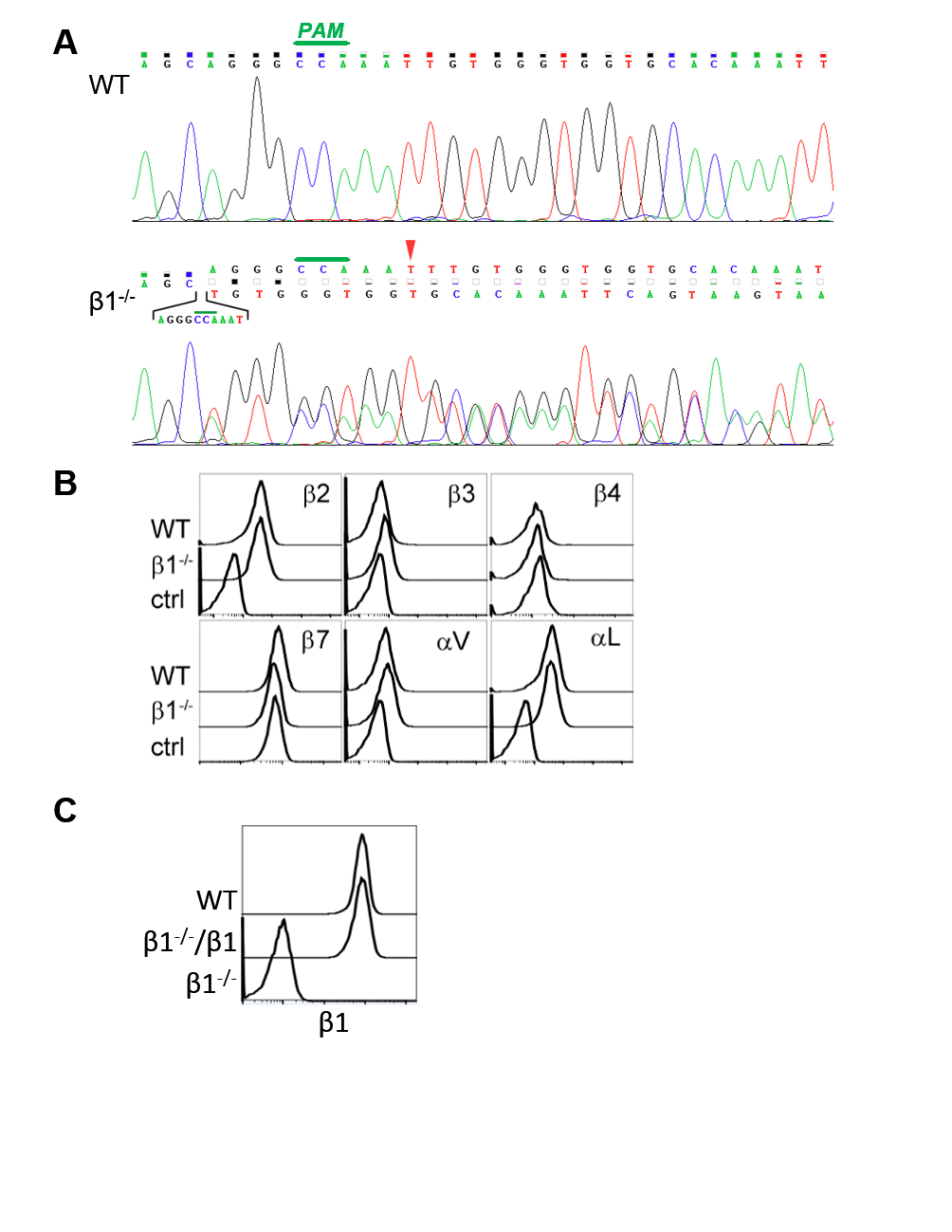

Supplement: Supplemental Figure 4 — CRISPR-Cas9 generation of integrin β1−/− Jurkat cells. (A) Sequencing of the ITGB1 genomic loci showing single nucleotide insertion (red arrow) in one allele and a 10 bp deletion (lower inset) in the other allele. PAM recognition motif indicated with green bar. Both frame shifted alleles encode for predicted truncated proteins of 64 and 65 amino acids resulting from premature termination codons. Wildtype β1 is 798 amino acids long. (B) Flow histograms for the indicated integrin expression in WT and β1−/− cells. (C) Flow histogram of WT, β1−/−, and β1−/−/β1 cells for integrin β1 expression. [file Image_4.TIF]
